# The Impact of Particulate Matter on Outdoor Activity and Mental Health: A Matching Approach

**DOI:** 10.3390/ijerph16162983

**Published:** 2019-08-19

**Authors:** Miyeon Jung, Daegon Cho, Kwangsoo Shin

**Affiliations:** 1 College of Business, Korea Advanced Institute of Science and Technology, 85 Hoegiro Dongdaemoon-gu, Seoul 02455, Korea; 2 Department of Bio-Medical Convergence, College of Medicine, Chungbuk National University, 1 Chungdae-ro, Seowin-gu, Cheongju-si 28644, Korea

**Keywords:** particulate matter, outdoor activity, mental health, propensity score

## Abstract

Exposure to air pollution affects human activity and health. Particularly, in Asian countries, the influence of particulate matter on humans has received wide attention. However, there is still a lack of research about the effects of particulate matter on human outdoor activities and mental health. Therefore, we aimed to explore the association between exposure to particulate matter with a diameter of less than 10 µm (PM10) and outdoor activity along with mental health in South Korea where issues caused by particulate matter increasingly have social and economic impacts. We examined this relationship by combining the physical and habitual factors of approximately 100,000 people in 2015 from the Korean National Health Survey. To measure each individual’s exposure to particulate matter, we computed the total hours exposed to a high PM10 concentration (>80 μg/m^3^) in a given district one month before the survey was conducted. After dividing all districts into six groups according to the exposed level of the high PM10, we applied the propensity score-weighting method to control for observable background characteristics. We then estimated the impact of the high PM10 on outdoor activity and mental health between the weighted individuals in each group. Our main findings suggest that the impact of PM10 on outdoor activity and stress shows an inverted-U shaped function, which is counterintuitive. Specifically, both outdoor activity and stress levels tend to be worsened when the exposure time to a high PM10 (>80 μg/m^3^) was more than 20 h. Related policy implications are discussed.

## 1. Introduction

Air pollution has caused significant health burdens worldwide [1,2]. The World Health Organization (WHO) estimates that three million premature deaths were attributable to ambient air pollution globally, in 2012. Since then, according to an annual report of the WHO, the number of deaths has rapidly grown to seven million premature deaths annually linked to air pollution, in 2014. Air pollution is expected to have a greater impact on Asian countries, especially those countries with rapid economic growth. Over the last decade, Asian countries have undergone substantial growth in urbanization, coupled with an increase in energy use [3,4]. Intense industrial activity and an unprecedented rise in motor vehicle usage have imposed severe environmental impacts in the region [5]. Therefore, air pollution has emerged as a significant threat to the environment and people’s quality of life. There is considerable evidence demonstrating that poor air quality has been wreaking havoc with the health of the populations in regions such as China, India, Malaysia, and Korea [6].

For example, China is suffering from socioeconomic costs that come as a result of the increases in particulate matter (PM) [7]. Northern China reached the level of PM2.5, which is about 40 times greater than the maximum level of PM2.5 allowed by the WHO [8]. The health consequences of such air pollution are enormous. A study, in 2010, found that premature deaths caused by air pollution in China were 1.2 million, accounting for almost 40% of the total premature deaths globally [9]. According to the Deutsche Bank report, China’s air quality will decline by 70% in 2025 [10]. 

China is not the only country with this problem. Addressing the increase in air pollution is also an urgent issue in South Korea. In 2014, according to the ”Annual Report of Air Quality 2016 of the Korea National Institute of Environmental Research”, the average level of PM10 concentration in Seoul was 1.4, 2.2, 2.4, and 2.8 times higher than that in Los Angeles, Paris, London, and Tokyo, respectively. Similarly, the average level of PM2.5 concentration in Seoul was 45 μg/m^3^, which is almost double as compared with that in Los Angeles and Tokyo [11]. In order to solve the problem of severe air pollution, the Korean government has actively engaged in negotiations with neighboring China and implemented radical public policies [12]. As the side effects of PM have been reported, finding the association between air pollution and health-related outcomes in Asian countries is a pivotal question to both academic researchers and policymakers.

**Figure 1:**
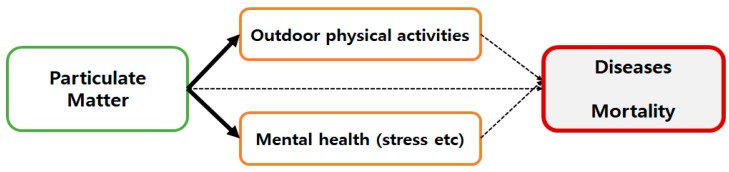
Research framework and the focus of this study. Note: An increase of particulate matter (PM) exerts an influence on outdoor activity and mental health. This is the locus of our study, as represented by the solid and bolded arrows. Outdoor physical activities and mental health affect a variety of diseases and mortality rates.

Despite the growing necessity for research, mediating variables between these diseases and PM, such as mental health or physical activity, have received little attention in prior studies (Figure 1), although outdoor activities and mental health are some of the most significant variables affecting the physical health of human beings [13]. Exercise is known to be highly beneficial to health, thus, when people do fewer physical activities, the probability of contracting disease becomes higher [13]. In addition, mental stress is the major cause of several diseases [14,15].

Previous studies, mostly performed in North American and European countries, have echoed that exposure to air pollution is associated with not only physical health [16] but also mental functioning. For example, Lavy et al. [17] and Weuve et al. [18] examined the impact of short-term exposure to ambient air pollution on cognitive performance, while Szyszkowicz et al. [19] examined the association between air pollution and depression. However, these studies have examined this association in Asian countries. In addition, the subjects in these studies [18,20] were mostly older women or patients that are not representative of the entire population.

Furthermore, previous studies have focused on the influence of air pollution with respect to severe mental illnesses (e.g., depression and suicidal tendencies) [21,22]. The effects of air pollution on the ordinary mental health of non-patients have rarely been studied. Given the preliminary findings that concluded poor air quality can give rise to feelings of annoyance and irritability in interpersonal relationships [23], it is necessary to investigate the association between air pollution and general mental health.

We also attempt to investigate the effect of PM on outdoor activity in this study. A decrease in outdoor activity may cause future health problems that can also result in the reduction of social productivity [24]. In this regard, it is not easy to address this association rigorously, because the associations can be highly confounded by several sociodemographic factors. The current study aims to help resolve the problem using a matching approach.

Specifically, we assess the impact of intensive exposure to PM10 on two focal dependent variables (the levels of outdoor activity and mental stress of individuals measured as the average walking days per week and self-reported mental health, respectively). These two variables and other detailed individual-level factors were identified from a large-scale population-representative national health survey conducted in 2015 in South Korea. We then combine this with the PM-level information that varies across regions. It is worth noting that by utilizing diverse and abundant features on an individual level, we can adequately match people across the exposure of PM10. By doing this, our study contributes to the literature regarding a comprehensive evaluation of the harmful effects driven by varying environmental conditions. Our main findings suggest, as anticipated, that high exposures to PM10 are associated with mental health deterioration and decreased outdoor activity in South Korea. However, the association does not seem to follow a simple linear pattern.

The rest of the paper is organized as follows. Section 2 describes the background for the study as well as a literature review. Section 3 describes the data and the method used. Section 4 presents our main findings and discussion. Section 5 presents the implications and concludes the paper.

## 2. Influence of Particulate Matter on Outdoor Activity and Mental Health

### 2.1. Particulate Matter and Outdoor Activity

The associations among air pollution, outdoor activity, and health outcomes have been examined in previous studies. Andersen et al. [25] looked at whether the effects of air pollution are moderated by physical activity in an urban setting and their results revealed that the increased respiratory uptake of air pollutants due to higher ventilation during physical activity amplifies the adverse effects of air pollution on health. Not surprisingly, people tend to reduce their outdoor activities in high air pollution environments. However, it may not be easy to rigorously measure this association due to various confounding factors (i.e., demographics and social status). Since air pollution is synchronized with regional productivity, individuals may, depending on their income, find themselves living in areas with bad air quality.

In terms of empirical methodology, most studies generally use the typical ordinary least squares regression model [26,27]. As far as mortality is concerned, the Poisson regression model or time series regression model with lagged variables is used to estimate the association between air pollution and mortality [28,29]. The negative impact of PM on health outcomes has been echoed in previous studies carried out in several contexts [30,31]. Moreover, there is consolidated evidence on the biological mechanisms linking the exposure to health damage, thereby substantiating the plausibility of the observed associations. The association between PM and physical health outcomes, such as mortality and disease, is also supported by both the epidemiological evidence and consistent empirical findings [32,33,34]. By contrast, the association between PM and human activity has not been widely examined. This may be due to the fact that human behaviors are difficult to measure constantly and accurately.

### 2.2. Particulate Matter and Mental Health

Cognitive performance, as a dimension of mental health, is critical to productivity in many occupations and is potentially linked to air pollution exposure. For example, one previous study evaluated this association by estimating the effect of PM exposure on academic test scores among Israeli high school students [17]. The authors found that exposure to PM exhibited a negative association on the test scores. Similarly, Weuve [18] found that long-term exposure to moderate PM levels in the United States is associated with cognitive declines in older women with respect to various cognitive aspects, such as verbal memory and attention.

Air pollution can affect not only cognitive performance but also emotions and feelings. In Canada, researchers reported the effects of air pollution on emergency department visits due to depression and suicide attempts [19]. Their results suggest that air pollution may lead to aggravated symptoms of depression among patients already experiencing it with a subsequent increase in emergency visits. Similarly, a study conducted in South Korea suggests that increased levels of PM10 aggravate depressive symptoms among the elderly [22].

In summary, previous studies have found that cognitive performance is negatively associated with PM pollution in students and older women. Additionally, other studies have found that depression and suicide attempts were associated with air pollution in hospital patients and the elderly. However, short-term exposure to air pollution can, in addition to depressive and functional illnesses, also affects people’s daily moods and general mental well-being.

In this regard, the focus of our study centers on the impact of PM concentrations on general mental health conditions. Although previous studies have examined the effects of air pollution on serious depression and cognitive functioning, there have been limited studies regarding the effects of PM on general mental well-being. Furthermore, previous studies have been restricted to homogenous groups of subjects, such as hospital patients and students, and have analyzed non-routine mental conditions, such as suicidal tendencies. It is, therefore, necessary to identify the effects of PM on general mental health by using national-level data that can represent the population.

Since we use data from a national-level survey, we can interpret the estimated effect with a more comprehensive perspective in terms of external validity. In addition, information on individual characteristics allows us to control these confounding factors when we estimate the effects of PM on outdoor activity and general mental health.

## 3. Method

### 3.1. Data Source and Descriptions

To accumulate a detailed population medical database for public health research, the Korea Centers for Disease Control and Prevention (KCDC) initiated the Korean Community Health Survey (KCHS) in 2008. It is the first nationwide survey to monitor and evaluate community health promotion and disease prevention programs. The standardized KCHS questionnaire covers a wide variety of health topics and the community-based cross-sectional survey is conducted by government-operated regional health centers.

Information on PM10 concentrations μg/m^3^ has been provided by Air Korea. This firm provides detailed data on the concentrations of PM per hour measured in abundant urban air monitoring networks, roadside air monitoring networks, and suburban air monitoring networks installed in districts across the country. We used district-level PM10 concentrations, which were further combined with KCHS data that uses the district-level addresses of individuals. Our final sample was comprised of 93,694 individuals living in 125 districts. Our dataset included each subject’s health status, diseases, lifestyle, education, and other individual characteristics.

### 3.2. Dependent Variables: Mental Health and Outdoor Walking Activity

The KCHS provides information on outdoor walking activity and self-reported mental health. The total number of walking days in recent weeks was measured with the question: “How many days did you walk for at least 10 min within a last week?” The question is one of the standard formats of The International Physical Activity Questionnaires (IPAQ). In terms of the validity of the question, many clinical studies have supported the correlation between self-reported IPAQ values with objective physical activity measures [35].

Self-assessed mental health was concerned with the question: “How much stress you feel in your everyday life within a last week?” The respondents indicated their recent mental stress on a level from 1 to 4: the lower the number, the higher the stress. Note that the period in which the survey was conducted was between August and October 2015. We assumed that the regional PM exposure level, calculated using the data in August, would be similar to patterns within a two month period.

### 3.3. Exposure Levels of High PM_10_

To calculate the individual varying indicators of high PM10 exposure, we used the residential district-level data of PM10, which is recorded on an hourly basis. For a more appropriate measurement, two components were considered, location and the amount of the exposure. The first involved the monitoring timestamp in a geographical information system (GIS) and the second involved the aggregation approach by using the raw data of the PM concentration.

**Figure 2:**
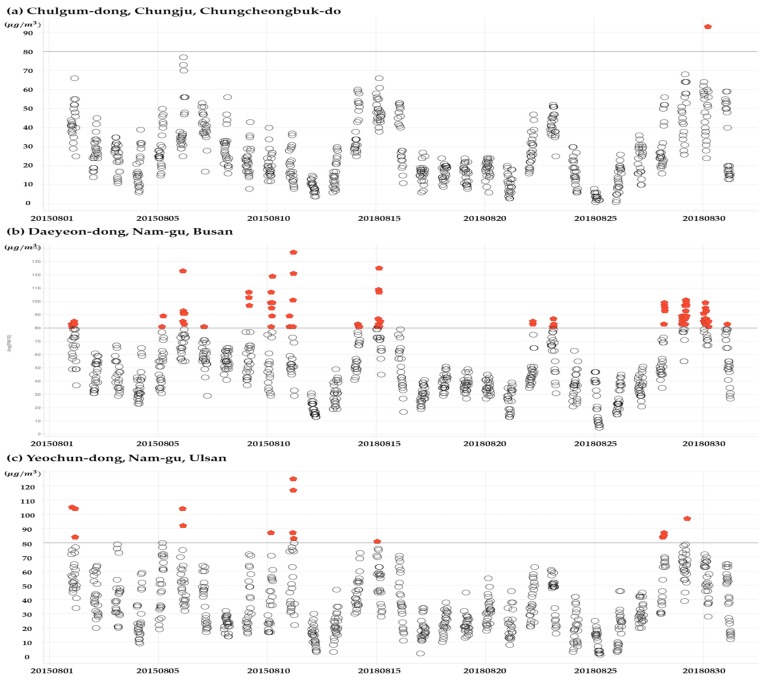
The method to calculate the total hour count of high PM10 exposure. Note: The *x*-axis denotes day, and the *y*-axis denotes the level of PM10. Each dot denotes an hourly level of PM10, so 24 dots are shown in each day. The red dots indicate that the level of PM for the time was higher than 80 µg/m^3^ between 1 August 2015, and 31 August 2015.

For the first issue, the temporal granularity of monitoring has shown wide variations in previous studies. The most dominant timestamp is the daily average or maximum level [36,37,38]. Only a few studies have used hour-level information [39,40,41,42]. We utilized this hour-level information to better indicate the ambient PM pollution to account for variations among different time points. As Figure 2 shows, the range in the concentration of PM10 is non-negligible (up to 45 µg/m^3^). Therefore, this study used PM10 monitoring information averaging pollution levels on an hourly basis. In addition, it is known that exposure to an extremely high PM concentration can have a greater direct effect on both mental health and outdoor activity, therefore, we used an additional measurement on PM10 concentration variance.

Secondly, the indicator of PM concentration needs to be identified. Most previous studies have used the mean annual concentrations of air pollutants on a city level [43,44]. However, using annual average levels is not adequate in our context. Following a previous study that involved the effects of ozone concentrations on farmer productivity [24], we excluded PM10 data during 12–5 a.m., and we used the data of 18 h/day for August.

The total observation comprised 558 h for each individual. We then calculated the total number of hours when the concentration of PM10 was >80 µg/m^3^, which is the threshold that assesses the “bad” PM10 level in South Korea. Finally, 125 districts in our data were equally divided into six groups based on the degree of exposure levels of high PM10 which was obtained by calculating the total number of hours with high PM10 concentrations.

We merged the two datasets (health survey and air quality) using district-level residential addresses. The addresses of individuals who responded to the health survey were matched with the air quality data for their district.

### 3.4. Statistical Analysis

To account for the potential endogeneity problem as much as possible, this study used an experimental setting by dividing the districts into six groups. Specifically, we compared the outcomes of treated (those who experienced a high concentration of PM10 and untreated individuals, assuming that the treatment (of a high concentration) was randomly assigned. In other words, living in a region with a high concentration of PM10 was considered as the treatment, which could not be randomly assigned and had to be related to other variables, such as family income or lifestyle. 

In this regard, Rubin [45] showed that the propensity score can be used to isolate the effect of a treatment on an outcome from other observed confounding factors that influence both the treatment assignment and outcomes. The propensity score can be used to reweigh comparison cases so that the distribution of their features matches between the treated and control groups. In this paper, we used the propensity score “weighting” approach calculated from a generalized boosted regression and we estimated the average treatment effect with five hypothetical treatment groups that were exposed to a higher PM10 level than the control (baseline, the lowest exposure) group.

We understand *f* (*x*|*t* = 1) to be the distribution of features for the treatment groups and *f* (*x*|*t* = 0) the distribution of features for the comparison groups. If the treatments were randomized, then we expect these two distributions to be similar. When they differ, we construct a weight, *w(x)*, therefore, the weight satisfies the following equations:

(1) f(x| t=1)= w(x)f(x), and f(x| t=0)= w(x)f(x).

The process of weighting can be easily explained by the simple task of multiplying the weight on one group and matching it with another group. If *f*(age = 60,sex = *F*|*t* = 1) = 0.10 and *f*(age = 60,sex = *F*|*t* = 0) = 0.05 (i.e., 10% of the treatment group and 5% of the control group are 60 year old women), then we needed to assign a weight of 2.0 to every 60 year old woman in the control group so that they have the same representation as in the treatment group. For multiple treatments, we typically apply Bayes Theorem to obtain the weights for all treatment groups. The detailed process of weighting has been described by Wooldridge [46,47] and McCaffrey et al. [48].

We divided the districts into six groups, based on the exposure levels as defined in Section 3.3. Among the 125 districts, approximately 20–21 districts belonged to each group. By setting Group 1 as the control group, we calculated the weight for all groups to remove any differences in the feature distribution. We weighted individuals in each group using 17 matching variables. Table 1 shows the definitions of the dependent (outcome) variables, independent (treatment) variables, and control (matching) variables in our study.

To analyze our data using the weights, we performed a propensity score-adjusted regression using R software. The package used was twang, which has a svyglm function for the purpose of average treatment effect estimation [49]. In this regard, the outdoor walking activity of a subject (*i*) in a region (*j*), where the PM exposure level of a region (*j*) has been assigned to one of the six groups (*K*), can be specified as follows:

(2) $$Number\;of\;Walking\_Days_{ij}\;=\;\alpha_{0}+\;\beta_{1-5}\;\left(Exposure\_Level\_of\_PM\_6scales^{K}{}_{j}\right)+\epsilon_{ij}\;.$$

Furthermore, the mental stress of a subject (*i*) in a region (*j*), where the PM exposure level of a region *j* has been assigned to one of the six groups (*K*), can be specified as follows:

(3) $$Mental\_Stress_{ij}=\;\alpha_{0}+\;\beta_{1-5}\;\left(Exposure\_Level\_of\_PM\_6scales^{K}{}_{j}\right)+\;\epsilon_{ij}$$

## 4. Results

### 4.1. Matching Variables of the Study Population and the PM_10_ Exposure Level

Table 2 summarizes the characteristics of 93,694 subjects across high PM10 concentration exposure levels, defined as the total number of hours experiencing high PM10 concentrations. The individual characteristics appeared mostly similar across quintiles of the PM10 exposure levels, however, there were some variations in the demographic and contextual factors. People exposed to higher PM10 concentration levels tended to be younger and live in regions with higher alcohol consumption and relatively sparse elderly populations. Since the individual characteristics varied across the six groups, the propensity scores to control for imbalances on the observed variables should be used. 

### 4.2. Assessment of Balance with Multiple Exposure Groups

**Figure 3:**
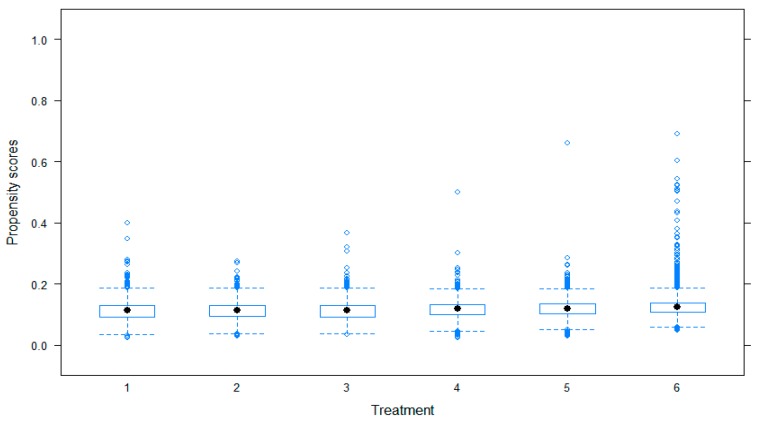
Propensity scores across groups.

It is important to confirm the diagnostic criteria for assessing the overall balance across multiple groups. The diagnostic is available by using the plotting function in the twang package in R software [49]. We report the distributions of the propensity score in Figure 3.

**Figure 4:**
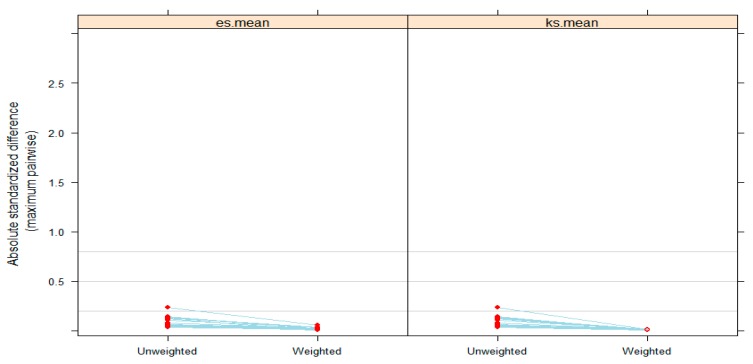
Absolute standardized differences across all groups.

As another graphical assessment of the balance, Figure 4 provides comparisons of the absolute standardized mean differences (ASMD) among the treatment groups on the matching variables before and after the weighting. As shown, after weighting, the maximum ASMD decreases for all matching variable covariates. The es.mean and ks.mean indicate the stopping rule in weight estimation algorithms. The stopping rules are defined by a balance metric for the covariates when the algorithm calculates the weights. The metric contains information of the difference between two univariate distributions of a pretreatment variable (that is, matching variables). The stopping rules in the twang R package use two balance metrics: absolute standardized bias (referred to as the effect size, ES) and the Kolmogorov–Smirnov (KS) statistic. The first part of the name of stopping rule means the balance metric (ES or KS), and the second part of the name specifies the method for summarizing values across all balance metrics for multiple matching variables. For example, the es.mean uses the effect size and summarizes values across variables with the mean and the ks.mean uses the KS statistics and also summarizes values using the mean across variables. As shown in Figure 4, the ks.mean stopping rule worked well, as it shows all hollow circles with a very small ASMD, which was used as the stopping rule in our weight estimation.

### 4.3. Effects on Mental Health and Outdoor Walking Activity

**Figure 5:**
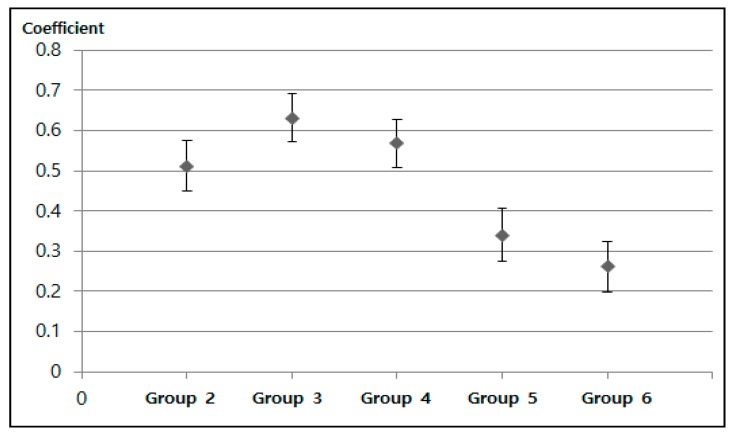
The estimated coefficient indicating the number of days that the participant walked at least 10 min per week. Note: The error bar indicates the 95% confidence interval of each coefficient.

After assessing the propensity score balance, we ran a regression model using the categorical variables of the six groups. The package twang serves as the estimation algorithm using this weight. Table 3 and Table 4 show the results regarding the effects of PM10 concentration on outdoor walking activities. In Figure 5, the figure roughly illustrates an inverted U-shape in the impacts of PM10 concentrations. Compared to Group 1, all groups from two to six show a higher number of walking days. This indicates that people who live in districts with very clean air environments walk less than others. However, after Group 3, the number of walking days tends to decrease. The inflection point is more than 20 h (the maximum value of Group 3) out of 558 h.

The exposure time of Group 6 is between 60 and 177 h (a total of 558 h), while Group 2′s exposure time is between 4 and 12 h. As shown in Table 3, when people are exposed to a high PM environment in more than 10% of their active time (i.e., about 60 h as in Group 6), they seem to reduce their outdoor activity by 0.25 days as compared with Group 2.

Table 4 shows the effect of high PM10 concentrations on mental health. When the exposure time was more than 20 h (the maximum value of Group 3), the coefficient starts to decrease, which means that mental stress worsens from Group 3.

**Figure 6:**
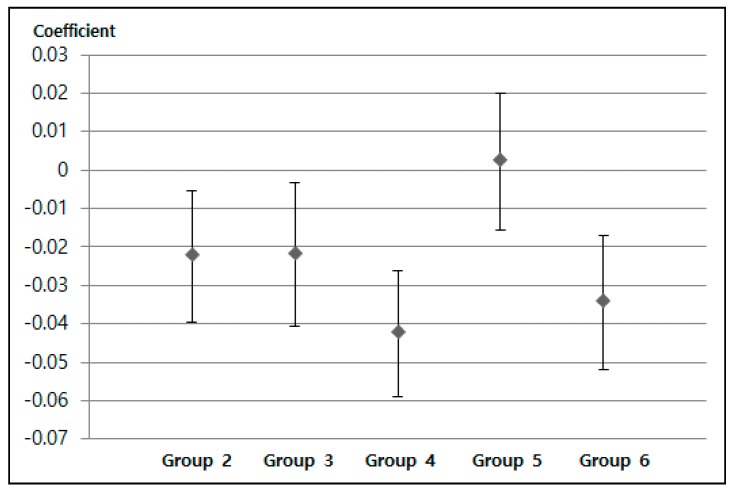
The estimated coefficient indicating self-reported mental stress (Likert 4 scale: 1, strong to 4, less likely) across the six groups. Note: The error bar indicates the 95% confidence interval of each coefficient.

Even though Group 5 showed an unexpected pattern, the overall trend shows an inverted U-shape with the inflection point at Group 3 (Figure 6). This pattern is also confirmed in our robustness check. Although we controlled for the effects of confounding variables, there could have been other uncontrolled confounding factors hindering our interpretation of the impact of PM concentration on mental stress. Nevertheless, we found evidence of a negative impact of PM exposure on mental stress levels in an inverted U shape.

To sum up, the number of days that the participants walked at least 10 min per week started to decrease when the exposure time was more than 20 h. Similarly, self-reported mental stress levels worsened when the exposure time was more than 20 h.

### 4.4. Robustness Checks

In this section, we present the following two robustness checks: (1) other specifications of regression equations and (2) alternative definitions of high PM10 exposure. First, we replace the previous five dummy variables indicating the belonged groups by the continuous variable of the exposure level. The continuous variable of the exposure level is calculated as the amount of exposure time with a high PM10 (>80 µg/m^3^), which is the same value used to divide the six groups. The range of exposure time is between 0 and 177 h. In this regard, the outdoor walking activity of a subject (*i*) in a region (*j*), where the PM exposure level of a region (*j*) has a continuous level, can be specified as follows:

(4) $$Number\;of\;Walking\_Days_{ij}=\;\alpha_{0}+\;\beta_{1}\;\left(highPM10\_hours_{j}\right)+\;\beta_{2}\;\left(highPM10\_hours_{j}{}^{2}\right)+\;\sum_{l=1}^{12}\gamma_{l}\;Individual\_Control_{i}+\sum_{l=1}^{124}\delta_{l}\;Region\_dummy_{j}\;+\;\epsilon_{ij}.$$

Furthermore, the mental stress of a subject (*i*) in a group (*j*), where the PM exposure level of a region j has a continuous level, can be specified as follows:

(5) $$Mental\_Stress_{ij}=\;\alpha_{0}+\;\beta_{1}\;\left(highPM10\_hours_{j}\right)+\;\beta_{2}\;\left(highPM10\_hours_{j}{}^{2}\right)+\;\sum_{l=1}^{12}\gamma_{l}\;Individual\_Control_{i}+\sum_{l=1}^{124}\delta_{l}\;Region\_dummy_{j}\;+\;\epsilon_{ij}$$

In Equations (4) and (5), the individual control variable includes age, sex, height, weight, ability to exercise, number of days in which breakfast is eaten, average time of sleeping, whether or not alcohol is consumed on regular basis, basic living support, number of household members, family income, and whether or not they engage in economic activities. We also included 124 dummies for each district except for one baseline city.

For Equations (4) and (5), we tested the inverted U-shape association between PM exposure levels, walking habits, and stress levels and investigated the inflection point based on the coefficients on linear and squared terms. We also tested the robustness of the inverted U-shape by using the threshold of a high PM10 as 100 µg/m^3^ instead of 80 µg/m^3^. We present the results with Equations (4) and (5) in Table 5.

The first column (1) in Table 5 shows the association between the time exposed to a high PM10 (>80 µg/m^3^) and walking days. We previously concluded that the number of walking days starts to decrease when the exposure time exceeds 20 h. As is evident in column one in Table 5, the coefficient of the linear term is positive and the coefficient of the squared term is negative, which means an inverted U-shape relation. Similarly, in the second column (2), we also find that the inverted U-shape remains in the association between exposure time and mental stress. 

**Figure 7:**
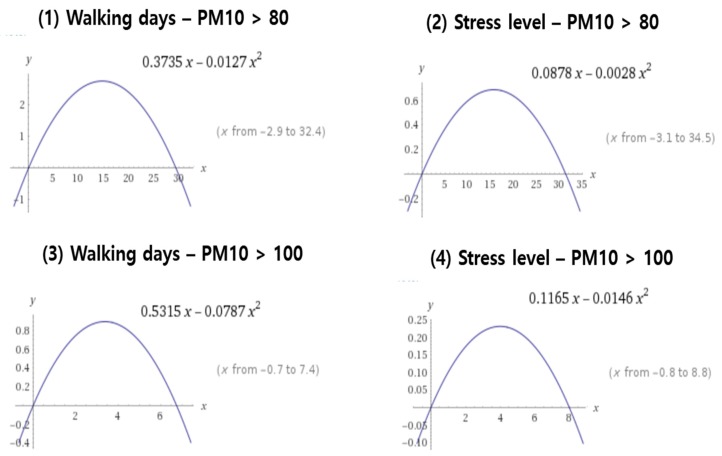
U-shape association between high PM10 exposure hours and focal outcomes. Note: The coefficients on x and x^2^ is from the coefficients on high PM10 hours and high PM10 hours X^2^ in Table 5.

In terms of the threshold, the coefficients *β*_1_ and *β*_2_ determine the inflection point in our specification. In Figure 7, the first and second graphs demonstrate that the inflection point for both outcomes is about 15 h, which is very similar to the 20 h deduced in our previous results. Therefore, the results confirmed that mental stress becomes worse and the number of walking days decreases after the exposure time to high PM10 (>80 µg/m^3^) exceeds 15 to 20 h.

Standard errors are in parenthesis. Coefficients on control variables were not reported.

The last two columns (3) and (4) in Table 5 are for the robustness check with a different threshold. It may be concerning that the threshold of 80 µg/m^3^ is not a proper criterion. The ”bad” criterion in South Korea is between 81 µg/m^3^ and 100 µg/m^3^, while the ”extremely bad” criterion (the worst level) is from 101 upwards. To examine the robustness of the inverted U-shape relation and to find a new inflection point under the more conservative criterion, we calculate the total exposure time using 100 µg/m^3^. As one can see from columns (3) and (4) in Table 5, the *β*_1_ and *β*_2_ remain as positive and negative signs, respectively, independent of the threshold. 

In addition, in columns (3) and (4) in Figure 7, these graphs demonstrate that the inflection points are about 3.5 h for walking days and 4 h for mental health. As expected, the new inflection points where mental health and walking days worsen are smaller than the previous inflection points under the threshold of 80 µg/m^3^. To conclude, people tend to decrease their outdoor walking activity and their mental health worsens when their exposure time to ”bad” PM10 levels (higher than 80 µg/m^3^) exceeds 20 h. If we defined the ”bad” PM10 as a value higher than 100 µg/m^3^, then people tend to decrease their outdoor walking activities and their mental health worsens if the exposure time to ”bad” PM10 exceeds about 4 h among a total of 558 h in a month. The exposure time is only about 1% of their active time, which means that a person’s outdoor activities and mental health are highly sensitive to ”extremely bad” PM10 concentration.

## 5. Conclusions

An assessment of the indirect costs of severe PM10 is difficult due to confounding real-world factors. Nevertheless, there should be more attention paid to the impacts that these factors have on human health. To this purpose, this study focused on the impact of PM concentration on mental health and outdoor activity. Using a large-scale population-representative national survey and novel approaches, we found an association between exposure to PM_10_ and outdoor walking activity and mental stress. Our findings suggest that when the PM exposure level exceeds a particular point (20 h of ”bad” PM10 and 4 h of ”extremely bad” PM10), people tend to invest less time in outdoor physical activities. Similarly, for mental health, PM exposure showed a negative impact as the exposure level increased after the same inflection point.

This study contributes to the existing literature on indirect social costs driven by high PM concentrations by showing the evident association between short-term exposure to air pollution and outdoor walking activities and mental stress levels on a national scale in South Korea. Since people in districts with a high exposure to PM_10_ concentrations demonstrated less walking activities and poor stress levels, policymakers need to consider such indirect health costs while performing a cost–benefit analysis of particulate matter prevention programs. If policymakers attempt to reduce the indirect health costs, this differential impact across regions can be considered.

Although this study used a unique approach and combined datasets that were not widely used in the previous work, our research has several limitations that can be addressed in future studies. First, our study estimated the effects of air pollution exposure on individuals using their addresses on a district level. Future studies can further investigate this association using addresses on a micro level, as well as their mobility information, to assess the impacts more precisely. Secondly, this study used the recent one-month pollution level from the survey period, and this may produce some differences from the actual outcome. Third, our study used the total hour count of high PM10 concentrations (>80 μg/m^3^) out of the total activity time in a month (558 h). Since we transformed the continuous PM10 concentration data into a binary one, the transformation may contribute to measurement error of high PM10 exposure level. Even though the use of the suggested indicator in this study is appropriate, future studies can further investigate whether the association is different with another precise measurement (e.g., showing time trend with hourly information). Fourth, there may be a further impact of exposure to PM2.5 on outdoor activity and mental health. Future studies can extend our work toward revealing this potential association.

## Figures and Tables

**Table 1:** Dependent, independent, and matching variables.

Variables			Definition
Dependent variables	Number of walking days		Number of days when he or she walked at least 10 minutes in the past week (from 0 to 7 days, including for commuting)
	Mental stress		The level of stress in daily life. (I feel very much a lot:1, I feel a lot:2, I feel a little:3, I hardly feel it:4)
Independent variables	Exposure level of PM		Six group indicators (1-6) divided by percent quintiles based on number of hours when PM10 concentration is higher than 80 μg/m^3^
Matching variables	Physical factors	Age	Age based on resident identification number (19–110 years old)
		Sex	Male:1, Female:2
		Height	The value of height in *cm*
		Weight	The value of weight in *kg*
		Ability to exercise	Exercise ability (1: I do not mind walking; 2: I have a little trouble walking; 3: I should be lying all day)
	Habitual factors	Number of days of intense physical activity	The number of days when he or she had at least 10 minutes of intense physical activity in the past week (from 0 to 7 days, such as running, hiking and cycling)
		Number of days of eating breakfast	Number of days when he or she had a breakfast in the past week (from 0 to 7 days)
		Average time of sleeping	Average time of sleeping per day (hour)
		Drinking or not up to now	Experience of drinking while living so far (Yes: 1, No: 0)
		Current drinking habit	Experience of drinking last one year (Yes: 1, No: 0)
	Socio-economic factors	Basic living support	Receiving basic living income or not (Yes: 1, Not now, but past recipients:2, No:3)
		Living together with dementia patients	Whether household is currently living with a dementia patient or not (Yes:1, No:0)
		Number of household members	Number of household members currently living together
		Family income	Average monthly income of household including wages, real estate income, interest, government supports in recent years (ask on 8 point scale)
		Economic activity	Whether he or she worked more than one hour with salary or worked for more than 18 hours as unpaid family workers in the past week (Yes:1, No:0)
		Owned car or not	Whether driving a car (Yes:1, No:0)
	Psychological factors	Perceived health condition	Think about her or his health (very good: 1, Good: 2, Usually: 3, Poor: 4, Very bad: 5)

**Table 2:** Matching variable across groups before weighting.

Characteristics		Number of Subjects	Exposure Level of High PM10 Concentration						
			Total	Quintile 1	Quintile 2	Quintile 3	Quintile 4	Quintile 5	Quintile 6
			(0–177)	(0–3)	(4–12)	(13–20)	(21–30)	(31–53)	(60–177)
Total		93,694	100.0 (%)	15.4	16.1	17.9	20.2	14.2	16.1
				14,451	15,073	16,783	18,918	13,350	15,119
Age	19–25	8864	9.5	8.4	9.1	9.9	9.8	9.7	9.7
	26–35	14,677	15.7	13.3	14.3	17.0	16.4	16.2	16.4
	36–45	19,200	20.5	19.8	19.8	20.4	20.6	20.8	21.6
	46–55	20,057	21.4	22.5	20.9	20.2	21.5	21.5	22.0
	56–65	15,968	17.0	17.9	18.1	17.4	16.2	17.4	15.6
	66–93	14,928	15.9	18.2	17.8	15.1	15.5	14.4	14.7
Sex	Male	47,211	50.4	51.9	49.4	50.3	49.9	50.8	50.2
	Female	46,483	49.6	48.1	50.6	49.7	50.1	49.2	49.8
Family Income	<500,000 won	4055	4.3	5.6	4.7	3.6	3.9	4.8	3.7
	500,000–1,000,000	8366	8.9	10.6	10.8	7.9	7.7	8.8	8.3
	1,000,000–2,000,000	13,988	14.9	16.9	16.7	14.5	13.8	14.4	13.7
	2,000,000–3,000,000	18,012	19.2	19.2	20.1	19.3	18.2	20.1	18.8
	3,000,000–4,000,000	17,676	18.9	18.0	17.8	20.4	18.1	18.7	20.2
	4,000,000–5,000,000	12,513	13.4	12.4	12.9	14.7	13.0	12.9	14.2
	5,000,000–6,000,000	7,707	8.2	6.9	7.8	8.0	8.8	8.3	9.4
	>6,000,000 won	11,377	12.1	10.4	9.2	11.7	16.6	12.0	11.8
Alcohol use (Drinking at least once in a year)		79,292	84.6	84.3	84.0	85.2	85.2	84.4	84.4
Vigorous Exercise (<3 days/week )		78,977	84.3	84.8	84.5	83.0	84.3	84.0	85.3
Height	≤150 cm	4163	4.4	4.7	4.9	4.0	4.5	4.5	4.1
	≤160 cm	28,029	29.9	30.1	30.9	29.7	29.4	29.7	29.9
	≤170 cm	36,058	38.5	38.8	38.4	38.2	38.5	38.6	38.4
	≤180 cm	22,622	24.1	23.5	23.1	25.0	24.4	24.2	24.5
	>180 cm	2822	3.0	2.8	2.7	3.1	3.2	3.0	3.2
Weight	≤50 kg	11,371	12.1	11.8	12.5	12.2	12.5	11.7	12.0
	≤60 kg	30,707	32.8	32.5	33.4	32.7	33.0	32.6	32.3
	≤70 kg	28,291	30.2	30.6	30.6	30.2	29.5	30.1	30.4
	≤80 kg	16,184	17.3	17.5	16.4	17.3	17.2	17.8	17.5
	>80 kg	7141	7.6	7.6	7.2	7.5	7.8	7.7	7.9
Ability to exercise	I do not mind walking	83,747	89.4	88.0	87.8	90.3	90.3	89.9	89.5
	I have a little trouble walking	9650	10.3	11.7	11.8	9.4	9.4	9.7	10.1
	I should be lying all day	297	0.3	0.3	0.3	0.3	0.3	0.4	0.3
Number of days of eating breakfast	0	12,745	13.6	11.6	12.6	13.5	14.2	15.2	14.5
	1	1842	2.0	1.6	1.9	2.3	2.0	2.2	1.8
	2	4129	4.4	3.8	4.1	4.9	4.6	4.3	4.6
	3	5446	5.8	4.9	5.8	6.2	5.8	5.7	6.6
	4	3101	3.3	3.0	3.4	3.6	3.2	3.2	3.4
	5	4060	4.3	3.7	4.3	4.5	4.6	4.4	4.5
	6	1524	1.6	1.2	1.7	1.9	1.9	1.4	1.5
	7	60,847	64.9	70.2	66.3	63.2	63.7	63.7	63.2
Average time of sleeping	one hour to 5 hours	15,610	16.7	16.0	16.7	16.8	16.8	16.9	16.7
	6 hours to 10 hours	77,932	83.2	83.8	83.2	83.0	83.0	83.0	83.1
	11 hours to 15 hours	149	0.2	0.2	0.1	0.2	0.2	0.2	0.2
Basic living support (Yes, or Having in the past, but not now)		3155	3.4	4.0	3.7	3.1	2.9	3.3	3.4
Living together with dementia patient		749	0.8	0.8	0.8	0.8	0.9	0.6	0.8
Number of household members	One person	9343	10.0	10.7	10.3	9.8	9.8	10.2	9.3
	Two persons	27,073	28.9	32.6	30.8	28.1	27.6	27.8	26.9
	3–4 persons household	47,570	50.8	47.0	49.4	51.7	52.0	51.0	53.1
	>4 persons	9708	10.4	9.7	9.5	10.5	10.7	11.1	10.7
Economic activity		61,558	65.7	65.4	65.3	65.1	65.0	67.1	66.6
Having a car		53,581	57.2	60.9	54.7	55.1	56.4	59.2	57.6
Perceived health condition	Very good	6756	7.2	6.2	6.4	7.4	8.2	7.3	7.5
	Good	32,491	34.7	34.1	34.6	35.8	35.5	33.8	33.8
	Moderate	40,569	43.3	43.0	43.1	43.2	42.5	44.5	43.8
	Poor	11,063	11.8	13.0	12.6	11.1	11.2	11.5	11.6
	Very bad	2815	3.0	3.7	3.3	2.5	2.6	2.8	3.2

**Table 3:** Effects of high PM_10_ concentration exposure levels on walking activity.

Variable	Average Number of Walking Days Per Week	
	Coefficient	Standard Deviation
Group 2	0.5118 ***	0.0314
Group 3	0.6312 ***	0.0302
Group 4	0.5681 ***	0.0295
Group 5	0.34 ***	0.0326
Group 6	0.2623 ***	0.0315
Constants	3.9192 ***	0.0229
Observations	93,694	

Note: *** *p*-value < 0.001.

**Table 4:** Effects of high PM_10_ concentration exposure levels on mental stress levels.

Variable	Perceived Mental Stress Level (Likert 4 Scale: 1, Strong to 4, Less Likely)	
	Coefficient	Standard Deviation
Group 2	−0.0224 **	0.0086
Group 3	−0.022 **	0.0094
Group 4	−0.0425 ***	0.0082
Group 5	0.0023	0.0089
Group 6	−0.0344 ***	0.0087
Constants	2.8812	0.0061
Observations	93,694	

Note: *** *p*-value < 0.001, and ** *p*-value < 0.01.

**Table 5:** Effects of high PM_10_ concentration exposure levels on walking days and mental stress levels: Continuous PM10 level with control variables.

Threshold	Hours with > 80 μg/m^3^		Hours with > 100 μg/m^3^	
Variable	(1) Perceived Mental Stress Level	(2) Number of Walking Days	(3) Perceived Mental Stress Level	(4) Number of Walking Days
high PM10 hours	0.3735 ***	0.0878 **	0.5315 ***	0.1165 **
	(0.0420)	(0.0431)	(0.0563)	(0.0579)
high PM10 hours^2	−0.0127 ***	−0.0028 **	−0.0787 ***	−0.0146 *
	(0.0013)	(0.0014)	(0.0076)	(0.0078)
Observations	93,694			

Note: *** *p*-value < 0.001, ** *p*-value < 0.01, and * *p*-value < 0.05. High PM10 hours^2 is a squared term of high PM10 hours.
